# Systematic Oxidative Stress Indexes Associated with the Prognosis in Patients with T Lymphoblastic Lymphoma/Leukemia

**DOI:** 10.1155/2022/2679154

**Published:** 2022-08-04

**Authors:** Liqin Ping, Yan Gao, Yanxia He, Xiaoxiao Wang, Bing Bai, Cheng Huang, Huiqiang Huang

**Affiliations:** Department of Medical Oncology, Sun Yat-Sen University Cancer Center, State Key Laboratory of Oncology in South China, Collaborative Innovation Center for Cancer Medicine, Guangzhou, Guangdong, China

## Abstract

**Background:**

T lymphoblastic lymphoma/leukemia (T-LBL/ALL) is an aggressive malignant tumor with 5-year overall survival (OS) rate reached 80% after high-dose chemotherapy. Due to the relatively low incidence of T-LBL/ALL, only a few risk factors have been identified. The occurrence and prognosis of malignant tumors are closely related to oxidative stress, but the prognostic relationship between T-LBL/ALL and systematic oxidative stress indexes has not been reported yet.

**Methods:**

A total of 258 T-LBL/ALL patients were retrospectively analyzed. The relationship between systematic oxidative stress indexes, such as creatinine (CRE), gamma-glutamyl transpeptidase (*γ*-GGT), albumin (ALB), alkaline phosphatase (ALP), fibrinogen (FBG), C-reactive protein (CRP) and total bilirubin (TBIL), and survival of T-LBL/ALL patients, was analyzed. The weight of indexes was used to calculate the patients' oxidative stress risk score. The independent prognostic value of oxidative stress risk grouping (OSRG) was analyzed.

**Results:**

Higher CRE, CRP, and lower ALB were associated with poorer OS in T-LBL/ALL patients. The OSRG established by CRE, ALB, and CRP was an independent prognostic factor for OS of T-LBL/ALL patients. Patients in the high-risk group were more likely to be patients older than 14 years old and patients with superior vena cava obstruction syndrome (SVCS), pleural effusion, pericardial effusion, and mediastinal mass.

**Conclusion:**

For OS in T-LBL/ALL patients, OSRG was observed as an independent prognostic factor, which provided a new idea for accurate prognostic prediction.

## 1. Introduction

T lymphoblastic lymphoma/leukemia (T-LBL/ALL) is a malignant tumor originating from progenitor T cells, accounting for only 2% of the lymphoma incidence. T-LBL/ALL mostly occurs in children and adolescents [1]. After intensive chemotherapy, the current 5-year overall survival (OS) rate can reach up to 80% [2]. However, the prognosis of refractory and recurrent T-LBL/ALL patients is very poor, and the 5-year OS is less than 20% [3]. Early detection of relapse may improve the outcome. Furthermore, high-dose chemotherapy can improve the survival and overall response rate (ORR), but treatment-related death (TRD) is one of the important factors affecting patients' outcomes [2]. Therefore, how to accurately predict the prognosis by performing risk stratification according to simple and feasible indicators before treatment is of great significance to guide treatment intensity and follow-up frequency. Due to relatively low incidence rate of T-LBL/ALL, few risk factors of T-LBL/ALL have been identified by the large samples.

Oxidative stress is considered an important factor in the occurrence and development of cancer, and it also affects the patient's prognosis [4, 5]. In the evolution of tumors, oxidative stress can cause dysregulation of tumor microenvironment metabolism and signaling pathways and lead to malignant transformation of cells [6]. Reactive oxygen species (ROS) may play an important role in T cell activation and signal transduction and may be associated with the development of T cell lymphoma [7]. However, in T cell lymphoma, tumor cells avoid ROS damage by down-regulating oxidative stress signaling pathways [8]. These evidences suggest that oxidative stress is closely related to T-LBL/ALL.

Previous studies had showed that hematologic indicators could reflect the status of systematic oxidative stress. Creatinine (CRE), C-reactive protein (CRP), and total bilirubin (TBIL) increased in oxidative stress mouse models and critically ill polytrauma patients, while decreasing after antioxidant treatment [9–12]. Albumin (ALB) is an important antioxidant in the body and is a marker commonly used to reflect oxidative stress levels [13]. Gamma-glutamyl transferase (*γ*-GGT), a commonly used indicator of oxidative stress, decreased after patients received antioxidant therapy [14]. Increased alkaline phosphatase (ALP) activity is associated with oxidative stress in colitis models [15]. Fibrinogen is the main plasma protein of oxidative modification and can be used as a marker to assess oxidative stress [16, 17]. Previously, we reported that systematic oxidative stress indexes are associated with the survival of breast cancer patients [18]. However, systematic oxidative stress indexes and prognosis of T-LBL/ALL have not been reported.

Therefore, the purpose of this study is to explore the relationship between systematic oxidative stress indexes and T-LBL/ALL. Afterward, it provides a new idea for predicting the prognosis of T-LBL/ALL, guiding the intensity of treatment and the frequency of follow-up.

## 2. Methods

### 2.1. Patients and Study Design

This study retrospectively enrolled patients who were pathologically diagnosed with T-LBL/ALL at Sun Yat-Sen University Cancer Center (SYSUCC) from January 2010 to December 2020. The inclusion criteria included as follows: (1) diagnosed as T-LBL/ALL by pathology in SYSUCC; (2) received standard antitumor therapy in SYSUCC; (3) patients underwent biochemical examination such as CRE, GGT, ALB, ALP, FBG, CRP, and TBIL within 3 days before chemotherapy; and (4) complete follow-up records. The exclusion criteria included as follows: (1) patients had received antitumor therapy before being transferred to our hospital; (2) absence of follow-up or biochemical information; (3) the liver and kidney dysfunction; and (4) accompanied by acute and chronic inflammatory diseases. The study is in line with the Declaration of Helsinki and the Ethics Committee of SYSUCC (identifier: B2022-155-01) as well.

### 2.2. Data Collection

We collected age, sex, Eastern Cooperative Oncology Group (ECOG) status, “B” symptoms, pathological diagnosis, clinical stage, superior vena cava obstruction syndrome (SVCS), central nervous system (CNS) involvement, mediastinal mass, pericardial effusion, pleural effusion, lactate dehydrogenase (LDH), and bone marrow (BM) biopsy examination of patients with T-LBL/ALL. The results of CRE, GGT, ALB, ALP, FBG, CRP, and TBIL were obtained within 3 days before the chemotherapy. An experienced pathologist at SYSUCC was requested to reassess the pathological diagnosis of the patients. The patient's age referred to the age of pathological diagnosis. The “B” symptoms referred to fever >38°C for more than 3 days, severe night sweats, and/or 10% weight loss without apparent cause in the last 6 months. The clinical stage was based on the Ann Arbor stage. Furthermore, mediastinal mass, pleural effusion, and pericardial effusion were also determined based on imaging findings. SVCS referred to edema of the upper limb, neck, face, and superficial varicose veins of the upper body caused by mediastinal mass. The biochemical test was analyzed by an automatic biochemical analyzer (Hitachi 7600 series, Tokyo, Japan). As for treatment, 205 patients (79.5%) received the BFM-90/95 regimen, in which pegaspargase was produced by Jiangsu Hengrui Pharmaceutical Co., Ltd. In addition, 13 patients (5.0%) received ECOG 2003 and 12 patients (4.7%) received hyper-CVAD/MA regimen. Another 28 patients (10.9%) received other regimens, such as GD 2008ALL and SCCLG-ALL-2016. After standard treatment, patients received a regular telephone or outpatient follow-up visits after the treatment. The last follow-up of the patients included in this study was conducted in June 2021.

### 2.3. Statistical Analysis

R software (version 4.0.2) and SPSS 24.0 were used to perform analysis of the data statistics of this study. The best cut-off values of GGT, ALB, CRP, TBIL, FBG, CRE, and BUN were determined by “survminer” package in “R” software. If the value is higher than the cut-off value, the value is 2; if the value is lower than the cut-off value, the value is 1. The univariate and multivariate Cox regression analyses were used to conclude the OS-independent prognostic factors. The correlation coefficient of systematic oxidative stress indexes was determined by the lowest AIC (Akaike information criterion) value [19]. Then, the systematic oxidative stress risk score of each patient was calculated. The Kaplan-Meier survival analysis was used to analyze the patients' survival. The time-dependent receiver operating characteristic (ROC) curve was used to compare the prognostic value of oxidative stress risk grouping (OSRG) and clinical prognostic indicators. To compare the correlations between variables, the Chi-square test with *p* value <0.05 was used to consider statistically significant in the two-tailed test.

## 3. Results

### 3.1. Information on Patients and Oxidative Stress Indicators

A total of 266 T-LBL/ALL patients at SYSUCC were retrospectively registered in our study. Out of 266, 8 patients died due to treatment-related complications. To ensure the prediction accuracy of this risk grouping, these 8 patients with TRD were excluded from the study. Of the 258 patients enrolled, 109 patients were younger, while 149 patients were older than 14 years. The patients' clinical features are presented in Table 1. Based on “Surv_cutpoint” analysis of R software, the optimal cut-offs of CRE, GGT, ALB, ALP, FBG, CRP, and TBIL were 44.6 *μ*mol/L, 21.2 U/L, 38.7 g/L, 106.7 U/L, 3.09 g/L, 12.28 mg/L, and 11.8 *μ*mol/L, respectively (Supplementary Figures 1(a)–1(g)).  Table 2 shows the distribution of systematic oxidative stress indexes of patients.

### 3.2. Calculation of Oxidative Stress Risk Score

**Figure 1:**
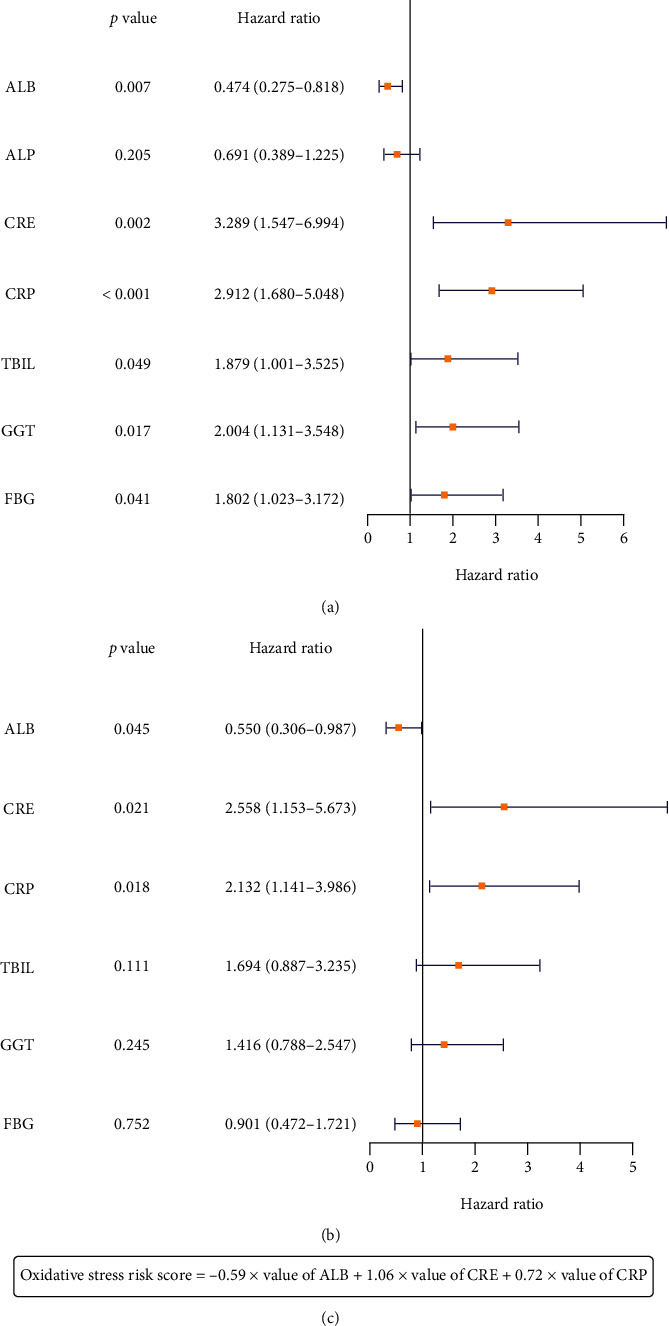
Establishment of oxidative stress risk score. The univariate (a) and multivariate (b) Cox regression analysis of oxidative stress indexes. (c) Calculation formula of oxidative stress risk score.

Conversion of CRE, GGT, ALB, ALP, FBG, CRP, and TBIL into dichotomy based on the cut-offs was done as described above. The values of “1” and “2” were used for scoring. The values were defined as 2 if it was higher than cut-offs, and 1 if they were lower. The univariate Cox regression analysis revealed that only ALP was not correlated with OS, while all other factors including CRE, GGT, ALB, FBG, CRP, and TBIL were correlated with OS (Figure 1(a)). In multivariate Cox regression analysis, the elevated CRP and CRE were related to worse outcomes in the T-LBL/ALL patients, while the elevated ALB was associated with better outcomes (Figure 1(b)). Then, to calculate the weight of oxidative stress indexes in the model, we calculated the correlation coefficient of indexes based on AIC. Oxidative stress risk score = -0.59 × value of ALB+1.06 × value of CRE+0.72 × value of CRP (Figure 1(c)). Then, OSRG of T-LBL/ALL patients was identified based on the median value of oxidative stress risk score. Patients with a risk score greater than the median value were in high-risk group, and patients with a risk score less than or equal to the median value were in low-risk group.

### 3.3. Relationship between OSRG and Clinical Features

**Figure 2:**
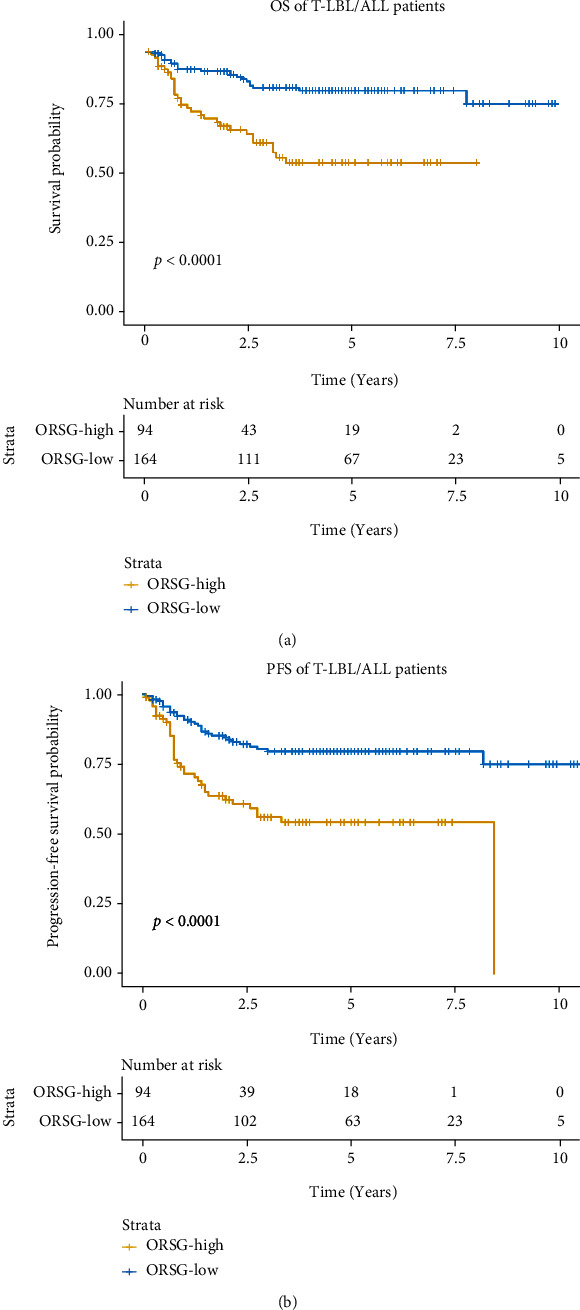
OSRG was related to prognosis in T-LBL/ALL patients. Kaplan-Meier curves revealed that the OS (a) and PFS (b) of high-OSRG patients were shorter than that of low-OSRG patients.

**Figure 3:**
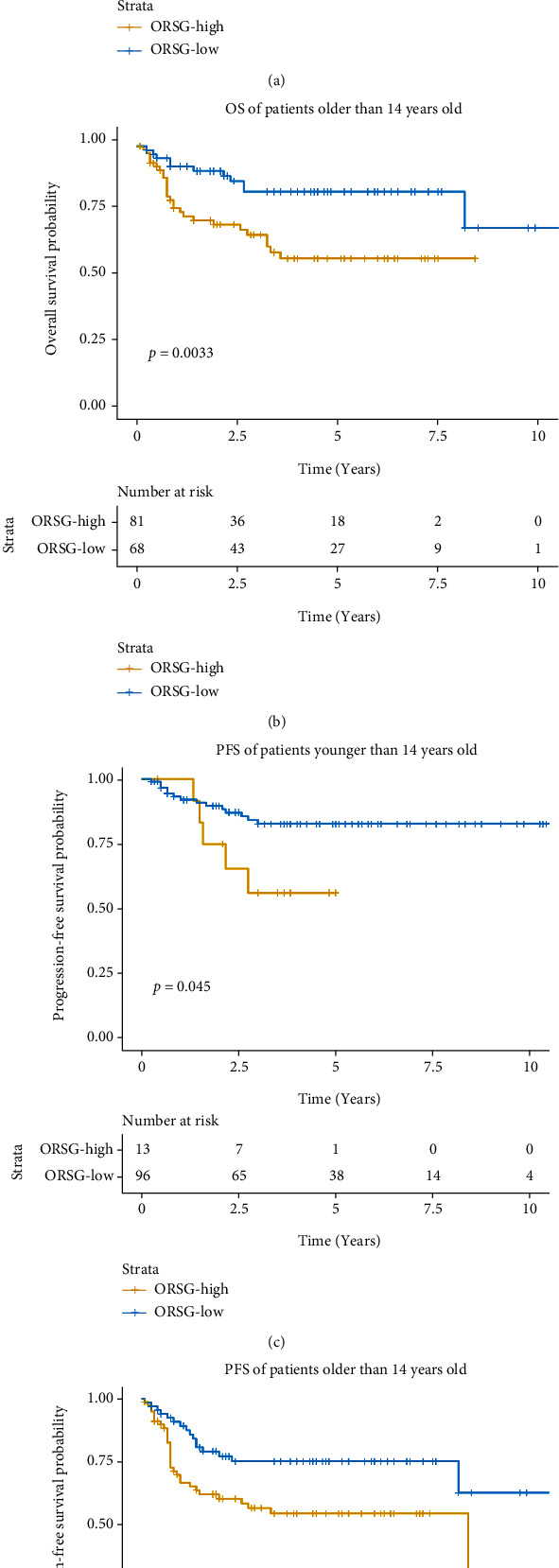
Subgroup survival analysis for T-LBL/ALL patients. (a) Kaplan-Meier analysis for the OS of patients younger than 14 years old. (b) Kaplan-Meier analysis for the OS of patients older than 14 years. (c) Kaplan-Meier analysis for the PFS of patients younger than 14 years old. (d) Kaplan-Meier analysis for the PFS of patients older than 14 years.

The group with high-risk was correlated with worse OS in the patients with T-LBL/ALL (Figure 2(a)). Meanwhile, we calculated the relationship between OSRG and progression-free survival (PFS). The results showed that the high-risk group had shorter PFS as compared to the low-risk group (Figure 2(b)). Similarly, in subgroup analysis, we found that OS and PFS were worse in the high-risk group than that in the low-risk group in both patients younger than 14 years old (Figures 3(a) and 3(c)) and patients older than 14 years old (Figures 3(b) and 3(d)). To further explore the relationship between OSRG and clinical features, we performed the Chi-square test, and the results revealed that the patients in high-risk group were more likely to be patients older than 14 years old, and patients with SVCS, pleural effusion, pericardial effusion, and mediastinal mass. At the same time, the complete response (CR) rate was higher in the low-risk group (Table 3).

### 3.4. Independent Prognostic Significance of OSRG

**Figure 4:**
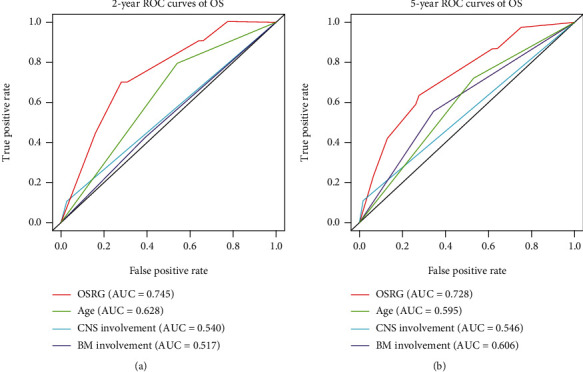
The predictive accuracy of OSRG is better than that of clinical prognostic indicators. The area under the ROC curves was used to compared the prognostic value of OSRG and clinical prognostic indicators for predicting 2-year OS (a) and 5-year OS (b).

In Kaplan-Meier survival analysis, the high-risk was associated with worse outcomes in T-LBL/ALL patients. To further explore its prognostic value, the univariate and multivariate Cox analysis was calculated for all the indicators that might affect the prognosis of the patients. The univariate Cox regression analysis showed that OSRG, age, CNS involvement, and BM involvement were correlated with the prognosis of the patients. The multivariate Cox regression analysis showed that OSRG, CNS involvement, and BM involvement were independent prognostic factors for T-LBL/ALL patients (Table 4). Further ROC curves revealed that the predictive accuracy of OSRG is better than factors of age, CNS involvement, and BM involvement. The area under the ROC curves was used to compare the prognostic value of OSRG and clinical prognostic indicators for predicting 2-year OS (Figure 4(a)) and 5-year OS (Figure 4(b)).

## 4. Discussion

Oxidative stress is closely related to cancer occurrence and cancer development as well as prognosis[4, 5, 20, 21]. Previously, we have reported that systematic oxidative stress indexes are related to the prognosis of breast cancer patients [18]. However, systematic oxidative stress indexes and prognosis of T-LBL/ALL have not been reported.

Albumin (ALB) is an important protein present in human plasma. In cancer patients with normal liver synthesis function, ALB is often associated with the patient's nutritional status and inflammation level [22]. It also has the effect of antioxidant stress [23]. Elevated ALB is often associated with better survival in cancer patients [24]. This study also showed that higher ALB was suggestive of longer OS in T-LBL/ALL patients. Creatinine (CRE) in the blood is derived from exogenous and endogenous. Endogenous CRE is the product of the metabolism of muscle tissue in the body. It has been shown in other studies that CRE is correlated to the level of oxidative stress in the body, which decreases after patients receive antioxidant treatment [12]. Higher CRE level is associated with a worse prognosis for patients with malignant tumor [25]. C-reactive protein (CRP) is an acute protein that reflects the level of inflammation in the body. Increased CRP can be observed in many malignant tumors and is associated with poor prognosis of tumor patients [26, 27].

The present investigation is the first to analyze the prognostic value of indicators of systematic oxidative stress on T-LBL/ALL. Among the 7 indicators of systematic oxidative stress, ALB, CRE, and CRP were independent prognostic factors for T-LBL/ALL patients. The multivariate Cox regression analysis showed that the OSRG established according to the weight of ALB, CRE, and CRP was an independent prognostic factor for the T-LBL/ALL patients. This study also confirmed that indicators of systematic oxidative stress is also associated with the prognosis of T-LBL/ALL. At the same time, we found the association between OSRG and clinical characteristics of T-LBL/ALL patients. The high-risk group was associated with older age, presence of SVCS, pleural effusion, pericardial effusion, and mediastinal mass. Additionally, the CR rate was also lower in the high-risk group than in the low-risk group. Patients with relapsed/refractory T-LBL/ALL have a poor prognosis, and early detection of disease progression is of great significance to patients. Further analysis showed that OSRG could predict PFS in patients, and patients in the high-risk group had a higher recurrence rate.

At present, there are few studies on the analysis of prognostic factors, which make this study important to prognosis prediction for T-LBL/ALL patients. In this study, the data of 258 patients with T-LBL/ALL were included and analyzed, confirming that the OSRG is an independent prognostic factor for T-LBL/ALL patients. T-LBL/ALL is one of the malignant tumors with an acceptable prognosis after intensive chemotherapy, but still, its TRD and disease progression are the factors causing the poor prognosis. This study can further provide clinicians with references for precise treatment and follow-up frequency for T-LBL/ALL patients.

However, the present study is a single-center retrospective study, and the specific mechanism of oxidative stress in tumor development and outcome is yet not clear. Due to the retrospective analysis, it failed to match the consistency of OSRG with the 2', 7'-dichlorofluorescin (DCFH) assay in detecting ROS. Furthermore, basic research and prospective study are needed in the future.

## Figures and Tables

**Table 1:** Clinical characteristics of study population.

Characteristics	Number (*n*)	Percentage (%)
*Sex*		
Male	196	76.0
Female	62	24.0
*Age*		
≤14	109	42.2
>14	149	57.8
*ECOG*		
<2	249	96.5
≥2	9	3.5
*B symptoms*		
Yes	56	21.7
No	202	78.3
*SVCS involvement*		
Yes	103	39.9
No	155	60.1
*Pleural effusion*		
Yes	96	37.2
No	162	62.8
*Pericardial effusion*		
Yes	53	20.5
No	205	79.5
*CNS involvement*		
Yes	10	3.9
No	248	96.1
*Mediastinal involvement*		
Yes	164	63.6
No	94	36.4
*BM involvement*		
Yes	102	39.5
No	156	60.5
*LDH status*		
Elevated	136	52.7
Normal	122	47.3
*Ann Arbor stage*		
I/II	18	7.0
III/IV	240	93.0
*ASCT/HSCT at CR*		
Yes	12	4.7
No	246	95.3
*Treatment response*		
CR	229	88.8
Non-CR	29	11.2

**Table 2:** oxidative stress indexes of study population.

Oxidative stress indexes	Number (*n*)	Percentage (%)
*CRE (μmol/L)*		
≤44.6	89	34.5
>44.6	169	65.5
*GGT (U/L)*		
≤21.2	132	51.2
>21.2	126	48.8
*ALB (g/L)*		
≤38.7	98	38.0
>38.7	160	62.0
*ALP (U/L)*		
≤106.7	154	59.7
>106.7	104	40.3
*FBG (g/L)*		
≤3.09	190	73.6
>3.09	68	26.4
*CRP (mg/L)*		
≤12.28	195	75.6
>12.28	63	24.4
*TBIL (μmol/L)*		
≤11.8	212	82.2
>11.8	46	17.8

**Table 3:** Relationship between OSRG and clinical characteristics.

Characteristics	OSRG-low (*n* = 164)		OSRG-high (*n* = 94)		*P* value
*Sex*					
Male	123	75.0	73	77.7	0.630
Female	41	25.0	21	22.3	
*Age*					
≤14	96	58.5	13	13.8	**0.000**
>14	68	41.5	81	86.2	
*ECOG*					
<2	156	95.1	93	98.9	0.108
≥2	8	4.9	1	1.1	
*B symptoms*					
Yes	32	19.5	24	25.5	0.259
No	132	80.5	70	74.5	
*SVCS*					
Yes	56	34.1	47	50.0	**0.012**
No	108	65.9	47	50.0	
*Pleural effusion*					
Yes	50	30.5	46	48.9	**0.003**
No	114	69.5	48	51.1	
*Pericardial effusion*					
Yes	17	10.4	36	38.3	**0.000**
No	147	89.6	58	61.7	
*CNS involvement*					
Yes	5	3.0	5	5.3	0.363
No	159	97.0	89	94.7	
*Mediastinal involvement*					
Yes	94	57.3	70	74.5	**0.006**
No	70	42.7	24	25.5	
*BM involvement*					
Yes	66	40.2	36	38.3	0.758
No	98	59.8	58	61.7	
*LDH status*					
Elevated	85	51.8	51	54.3	0.707
Normal	79	48.2	43	45.7	
*Ann Arbor stage*					
I/II	15	9.1	3	3.2	0.071
III/IV	149	90.9	91	96.8	
*ASCT/HSCT at CR*					
Yes	6	3.7	6	6.4	0.317
No	158	96.3	88	93.6	
*Treatment response*					
CR	152	92.7	77	81.9	**0.008**
Non-CR	12	7.3	17	18.1	

**Table 4:** Results of the univariate and multivariate Cox regression analysis for OS.

Variables	Univariate Cox analysis		Multivariate Cox analysis	
	HR (95% CI)	*P* value	HR (95% CI)	*P* value
*Sex*				
Male	Reference	0.905		
Female	0.961 (0.504-1.833)			
*Age*				
≤14	Reference	*0.008*	Reference	0.102
>14	2.305 (1.248-4.258)		1.797 (0.890-3.628)	
*ECOG*				
<2	Reference	0.771		
≥2	1.234 (0.300-5.079)			
*B symptoms*				
No	Reference	0.582		
Yes	1.193(0.637-2.234)			
*SVCS involvement*				
No	Reference	0.224		
Yes	1.402 (0.813-2.415)			
*Pleural effusion*				
No	Reference	0.492		
Yes	1.215 (0.698-2.115)			
*Pericardial effusion*				
No	Reference	0.131		
Yes	1.604 (0.868-2.961)			
*CNS involvement*				
No	Reference	*<0.001*	Reference	*<0.001*
Yes	4.395 (1.872-10.322)		4.367 (1.845-10.339)	
*Mediastinal involvement*				
No	Reference	0.212		
Yes	1.466(0.804-2.673)			
*BM involvement*				
No	Reference	*0.030*	Reference	*0.010*
Yes	1.834 (1.060-3.173)		2.095 (1.198-3.666)	
*LDH status*				
Elevated	Reference	0.277		
Normal	1.360 (0.782-2.368)			
*Ann Arbor stage*				
I/II	Reference	0.698		
III/IV	0.832 (0.329-2.105)			
*ASCT/HSCT at CR*				
No	Reference	0.735		
Yes	1.244 (0.381-3.930)			
*OSRG*				
Low	Reference	*<0.001*	Reference	*0.002*
High	3.377 (1.929-5.911)		2.759 (1.470-5.180)	

## Data Availability

These data are available by individual application to corresponding author.

## Supplementary Materials

Supplementary materials Supplementary Figure 1. Identification of optimal cut-off values of oxidative stress indexes. Supplementary Figure 1. Identification of optimal cut-off values of oxidative stress indexes. The optimal cut-off values of ALB (A), ALP (B), CRE (C), CRP (D), TBIL (E), GGT (F), and FBG (G) were identified.
