# Supplementary figures and images for: Systematic Oxidative Stress Indexes Associated with the Prognosis in Patients with T Lymphoblastic Lymphoma/Leukemia
Source: Oxid Med Cell Longev. 2022 Aug 4;2022:2679154. doi: 10.1155/2022/2679154 (PMC9371838; doi:10.1155/2022/2679154)

**A**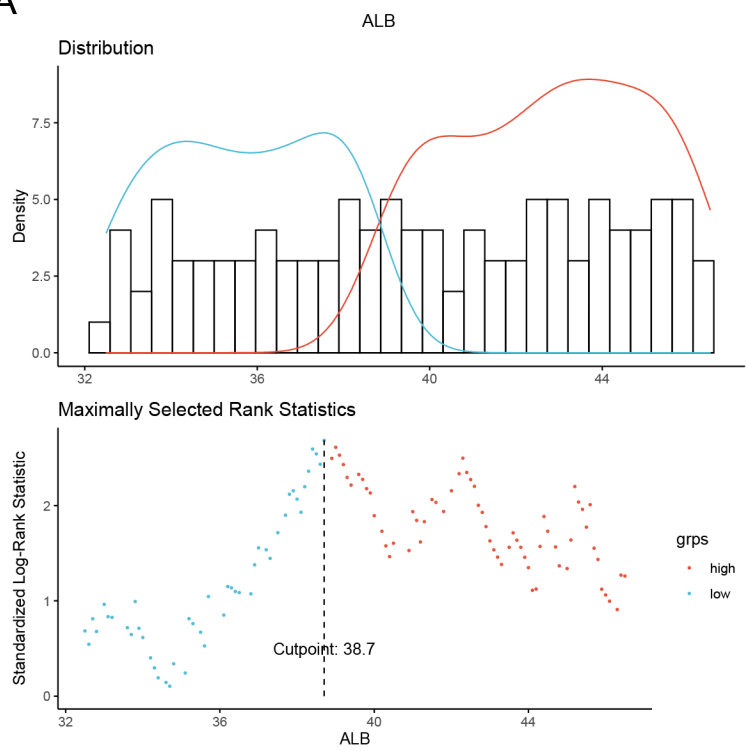**B**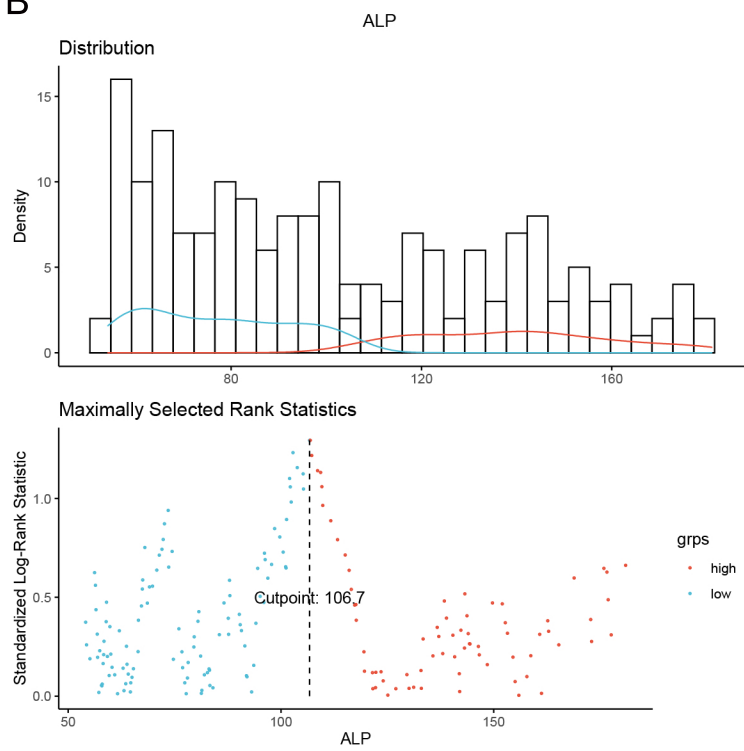**C**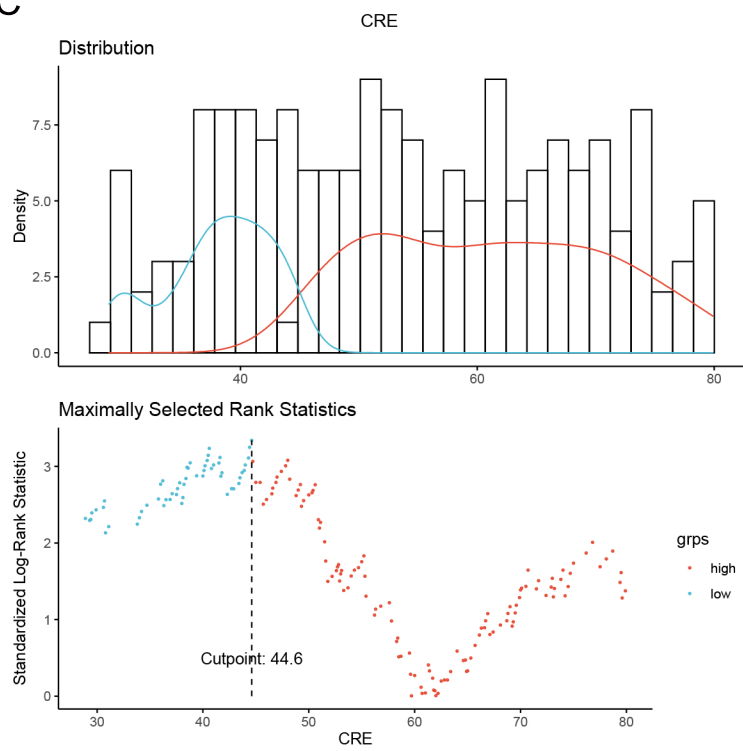**D**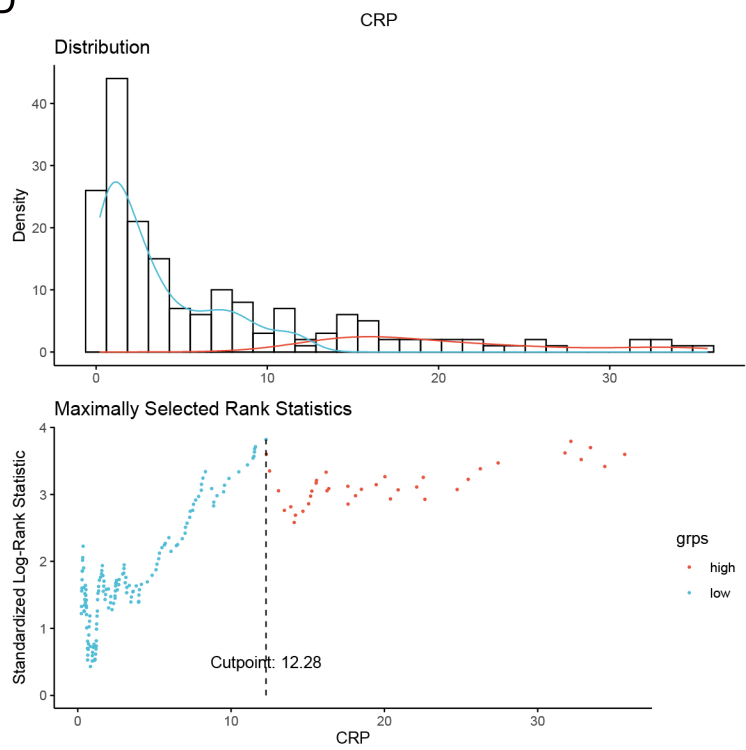**E**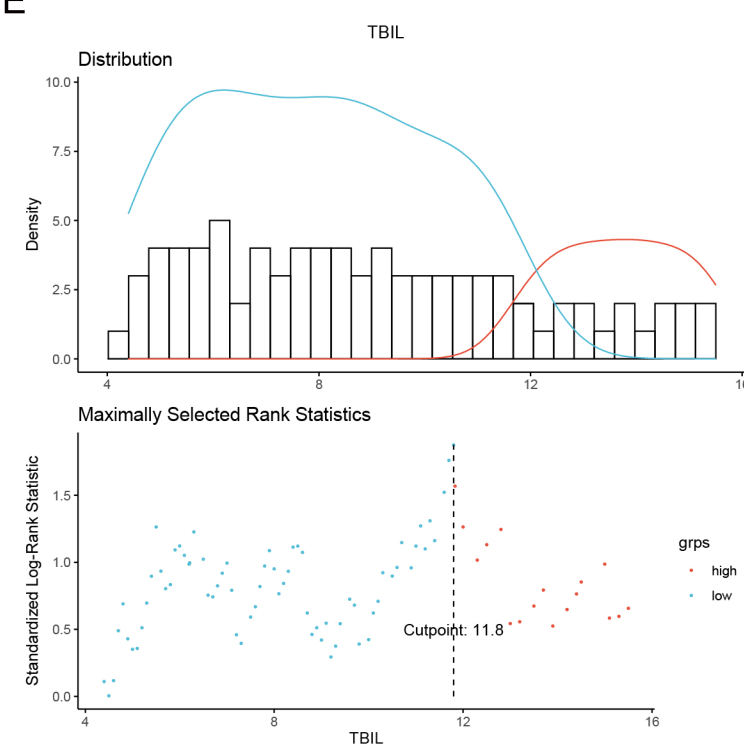**F**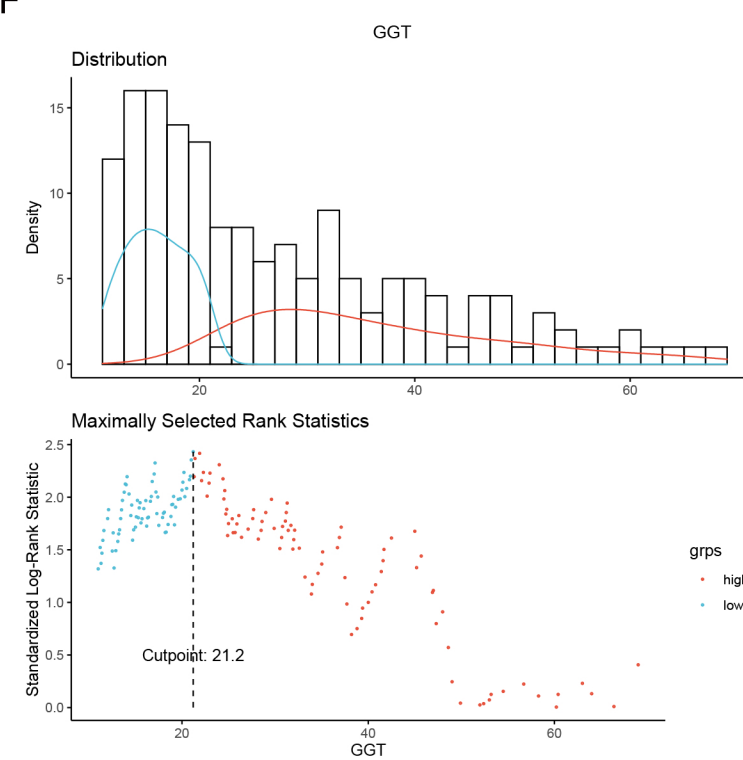**G**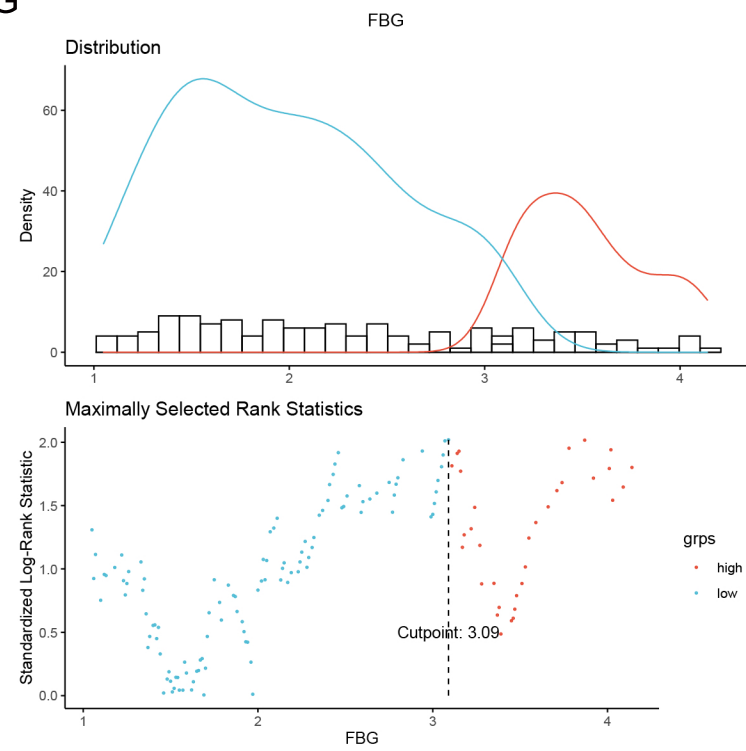

Supplement: Supplementary materials — Supplementary Figure 1. Identification of optimal cut-off values of oxidative stress indexes. Supplementary Figure 1. Identification of optimal cut-off values of oxidative stress indexes. The optimal cut-off values of ALB (A), ALP (B), CRE (C), CRP (D), TBIL (E), GGT (F), and FBG (G) were identified. [file 2679154.f1.pdf]
